# A call for healing and unity

**DOI:** 10.1128/mbio.00624-25

**Published:** 2025-02-27

**Authors:** Patrick D. Schloss

**Affiliations:** 1 Department of Microbiology & Immunology, University of Michigan 242912 , Ann Arbor, Michigan, USA

**Keywords:** diversity, equity, inclusion, science

## EDITORIAL

Executive orders signed since 20 January 2025 aim to end programs intended to diversify the scientific workforce via equitable and inclusive practices and processes; cut federal funding for research and innovation; and stifle scientific communications. They represent a stunning reversal of policies that have been the bedrock of the U.S. scientific research enterprise and its leadership in the world, and therefore not surprisingly they have been met with a number of legal challenges. Some of the executive orders have been blocked by temporary restraining orders, and others are pending judicial review.

These actions run counter to a declaration made over four decades ago by the U.S. Congress that “the highest quality of science and engineering over the long-term requires substantial support, from currently available research and educational funds, for increased participation in science and engineering by women, minorities, veterans and persons with disabilities” (1). In fact, an argument can be made that it is not possible to achieve scientific excellence without diversity, for lack of diversity provides prima facie evidence of structural problems in the scientific enterprise that preclude full participation by all groups in the society it serves. Given that scientific and mathematical talent is found in all human groups, lack of diversity in science means that highly talented individuals are not represented or participating in the enterprise. Thus, the full potential of humanity is not engaged. And as has been noted for corporate entities, diversity is a strength and correlates with better performance and outcomes (2).

Scientists at all levels have looked towards their institutions and professional scientific societies for leadership, solace, and answers in this time of uncertainty and upheaval. Yet, some of those administration actions also targeted these same societies and institutions, leading them to examine their vulnerabilities to not only protect their mission but also their constituents and stakeholders, and the advances they have made. As the chair of the ASM Journals Committee, I can attest that the ASM Journals program stands firm by this mission of the ASM and remains as committed as ever to the core values of ASM and to ensuring its success and our collective progress.

ASM temporarily removed some of its webpages for review as part of this effort to be cautious and ensure compliance with legal obligations. While this was important to ensure continuation of federal funding for ASM’s work, it understandably raised concerns among the microbial sciences community. To be clear, no journal articles were ever removed or hidden during this process. In the absence of advance communication, ASM’s actions were perceived as premature, particularly before clear directives from the government were developed. Some community members felt, understandably, abandoned or betrayed by the removal of these webpages. ASM leadership responded in a statement that this was not their intention, immediately worked to make amends among its community, and followed up with a town hall in which the leadership apologized for its misstep and pledged to be more mindful moving forward (3). It was obvious that members of the microbial sciences field felt pain due to ASM’s actions. But I am compelled to send a message calling for unity among the microbial sciences and a renewal of our commitment to support our diverse community of scientists, especially those historically excluded from science.

It is important to remember ASM’s longstanding commitment and record of addressing these issues. One critical example is ABRCMS, which has also supported all communities in science, technology, engineering, and mathematics. In addition, ASM has always supported its journals and provides editorial independence as we have created various avenues for diverse voices to be heard. This includes the *mSystems* series Social Equity and Disparities in Microbial Exposure (4), *mSphere’s* mSphere of Influence articles (5), the *Microbiology Spectrum* series on Diagnostic Testing and Laboratory Equity in Clinical Microbiology (6), papers that have examined disparities in scientific publishing (7), the ASM Journals statement supporting Black microbiologists (8), open calls that several of our journals have used to identify and recruit new editors to scientific publishing (9), and other editorials and articles submitted by ASM members (e.g., references 9–12). ASM’s journals remain committed to amplifying these voices and will continue to solicit, publish, and promote articles representative of the global microbiology community of scientists.

Now more than ever we need to lean into our community for support and gain strength in numbers. As a collective with shared ideals, we are well equipped to both withstand and respond to these challenges and any others that may follow. Everyone must have a voice in charting the path forward. Together, we must remain committed to our core values and take solace in the strength and intention of the scientific community. The ASM community demonstrates these core values in the work we all do every day and in its decades-long dedication to and advocacy for the entire scientific community. If instead we allow fractures to be sown among us, then we are weaker because we are distracted from our shared mission. Indeed, the scientific community must shore itself against divisions and focus as one on our mission—advancing and protecting science, the community of scientists, and the values we seek to uphold. In this way, we can best serve the public, who rely on and support our efforts for their health and well-being.

## EDITOR'S NOTE

**Ed. Note:** This editorial is being published by the following ASM journals: *Antimicrobial Agents and Chemotherapy*, *Applied and Environmental Microbiology*, *ASM Case Reports*, *Clinical Microbiology Reviews*, *Infection and Immunity*, *Journal of Bacteriology*, *Journal of Clinical Microbiology*, *Journal of Microbiology and Biology Education*, *Journal of Virology*, *Microbiology and Molecular Biology Reviews*, *Microbiology Resource Announcements*, *Microbiology Spectrum*, *mBio*, *mSphere*, and *mSystems*.
